# Development and Validation of a Nomogram Based on Motoric Cognitive Risk Syndrome for Cognitive Impairment

**DOI:** 10.3389/fnagi.2021.618833

**Published:** 2021-04-16

**Authors:** Yong Liu, Kai Wei, Xinyi Cao, Lijuan Jiang, Nannan Gu, Lei Feng, Chunbo Li

**Affiliations:** ^1^Shanghai Key Laboratory of Psychotic Disorders, Shanghai Mental Health Center, Shanghai Jiao Tong University School of Medicine, Shanghai, China; ^2^Clinical Neurocognitive Research Center, Shanghai Mental Health Center, Shanghai Jiao Tong University School of Medicine, Shanghai, China; ^3^Department of Psychological Medicine, Yong Loo Lin School of Medicine, National University of Singapore, Singapore, Singapore; ^4^Center for Excellence in Brain Science and Intelligence Technology (CEBSIT), Chinese Academy of Sciences, Shanghai, China; ^5^Institute of Psychology and Behavioral Science, Shanghai Jiao Tong University, Shanghai, China; ^6^Brain Science and Technology Research Center, Shanghai Jiao Tong University, Shanghai, China

**Keywords:** motoric cognitive risk syndrome, slow gait, subjective cognitive decline, frailty, cognitive impairment, nomogram

## Abstract

**Objective:**

To develop and validate a prediction nomogram based on motoric cognitive risk syndrome for cognitive impairment in healthy older adults.

**Methods:**

Using two longitudinal cohorts of participants (aged ≥ 60 years) with 4-year follow-up, we developed (*n* = 1,177) and validated (*n* = 2,076) a prediction nomogram. LASSO (least absolute shrinkage and selection operator) regression model and multivariable Cox regression analysis were used for variable selection and for developing the prediction model, respectively. The performance of the nomogram was assessed with respect to its calibration, discrimination, and clinical usefulness.

**Results:**

The individualized prediction nomogram was assessed based on the following: motoric cognitive risk syndrome, education, gender, baseline cognition, and age. The model showed good discrimination [Harrell’s concordance index (C-index) of 0.814; 95% confidence interval, 0.782–0.835] and good calibration. Comparable results were also seen in the validation cohort, which includes good discrimination (C-index, 0.772; 95% confidence interval, 0.776–0.818) and good calibration. Decision curve analysis demonstrated that the prediction nomogram was clinically useful.

**Conclusion:**

This prediction nomogram provides a practical tool with all necessary predictors, which are accessible to practitioners. It can be used to estimate the risk of cognitive impairment in healthy older adults.

## Introduction

The prevalence of age-related cognitive disorders, such as cognitive impairment and dementia for which few treatments are available, has been on the rise along with increasing world population, and these cognitive disorders have been shown to be independently associated with mortality in older people (Perna et al., 2015; Fogg et al., 2019). It is of paramount importance for clinicians to monitor cognitive change in older adults for timely taking steps to delay or reverse these conditions (Brasure et al., 2018; Gates et al., 2019). Research on diagnosing these diseases at their earliest stages via risk factors may be the most successful strategy for individualizing cognition monitoring. Previous work had shown that physiological risk factors [i.e., slow gait (SG) speed (Chou et al., 2019), low grip strength (Chou et al., 2019; Liu et al., 2019), poor standing balance (Rolland et al., 2009), diabetes (Moran et al., 2019), and sustained hypertension (Walker et al., 2019)], behavioral risk factors (i.e., low physical activity; Brasure et al., 2018; Müller et al., 2018; Palta et al., 2019), demographic risk factors (i.e., age and gender; Nebel et al., 2018), environmental risk factors (i.e., air pollution; Paul et al., 2019), and genetic risk factors (Chang et al., 2018) are linked to cognitive impairment and dementia. However, only one domain of the above risk factors is poorly predictive of cognitive status, and their applicability and sensitivity in routine clinical practice are not convincing. For example, the concept termed “frailty” combines most physical performance tests and is considered as an early stage of disability (Fried et al., 2001). In comparison to participants free of frailty or cognitive impairment, the pooled hazard ratios (HRs) for dementia are the following: 3.83 for isolated cognitive impairment, 1.47 for isolated physical frailty, and 5.36 for their co-occurrence. The co-occurrence of cognitive impairment and physical frailty is a clinical marker of incident dementia (Grande et al., 2019). Furthermore, a syndrome called “motoric cognitive risk syndrome” (MCR), which is a combination of physiological risk factors (SG) and dementia risk assessments [subjective cognitive decline (SCD)], can be also used to identify older individuals at risk of cognitive impairment (Verghese et al., 2014). MCR is a novel approach that can be used even in resource poor settings and has incremental predictive validity for dementia as compared to its individual components and even after accounting for overlap with mild cognitive impairment (Verghese et al., 2014). However, no one method, including MCR, fulfills all requirements to be considered as a reference today.

Thus, the combined analysis of a prediction model based on a panel of risk factors, rather than individual analyses, may be the most promising approach to identify cognitive disorders. Nomograms, a pictorial representation of a complex mathematical formula (Grimes, 2008), use various variables to determine a prediction model that generates a probability of a clinical event, such as dementia or death. Several prediction nomogram models have been developed to predict dementia (Downer et al., 2016) or cognitive impairment (Zhou et al., 2020), but these models were mostly based on demographic risk factors, behavioral risk factors, and comorbidities. So far, prediction nomogram models are lacking that include information on markers that reflect the underlying disease process, especially in its early stages. Such markers include MCR or others. Although various kinds of risk factors have been considered and demonstrated, an optimal approach based on MCR or others as a predictive signature has yet to be developed. Therefore, this study aimed to develop and validate a prediction nomogram that incorporates MCR and other risk factors for individual preoperative prediction of cognitive impairment in healthy older adults.

## Materials and Methods

### Development Cohort

Participants with baseline variables of interest from June 2011 to January 2016 were identified (*n* = 15,703) from the China Health and Retirement Longitudinal Study (CHARLS) (Zhao et al., 2014). Those who had missing values in one or more variables of interest (i.e., grip strength, gait speed, standing balance, chair stands, physical activity, SCD, weight loss, exhaustion) (*n* = 12,697), those 60 years or younger (*n* = 52), those who had stroke or memory-related diseases (*n* = 322) and disability (*n* = 1,040) were excluded from the analysis. In the remaining 1,592 participants, we further excluded those who were lost to follow-up (*n* = 154) and did not complete the cognitive test at baseline or follow-up (*n* = 96). We also classified participants as cognitive impairment if they were in the lowest 10% of the distribution of cognitive performance; thus, 165 participants with cognitive impairment at baseline were further excluded, as well. This resulted in a total 1,177 adults 60 years or older who received at least one follow-up. The process for selecting participants in the development cohort is shown in Supplementary Figure 1. Each participant signed a written informed consent form before taking part in the survey. Ethics approval for data collection in CHARLS was obtained from the Biomedical Ethics Review Committee of Peking University (IRB00001052-11015).

### Validation Cohort

To ensure the adoption of best practices and international comparability or results, CHARLS is harmonized with leading international research studies in the Health and Retirement Study (HRS) (Sonnega et al., 2014) model. As a result, we used the cohorts in HRS for external validation. The validation (Supplementary Figure 2) cohort from the HRS consisted of 2,076 participants from April 2012 to April 2016 using the same criteria in the development cohort. This assessed whether the prediction nomogram could be used to predict cognitive impairment risk for healthy participants in the United States. HRS, which has been fielded every 2 years since 1992, is a nationally representative longitudinal survey that builds understanding of aging. To be consistent for validation, curation of HRS data followed the same process that was done for CHARLS.

### Potential Predictors

The following 35 potential predictive variables were collected: demographic variables (8 variables), health status (16 variables), frailty and other physical performance test (8 variables), SCD, MCR, and baseline global cognition.

#### Demographic Variables

Demographic variables included age, gender, marital status, educational attainment, residence, body mass index (BMI), smoking status, and drinking habits. Gender was defined either as male or female. Marital status was defined as married if the participant was currently married and living with a spouse, or single if the participant was currently separated, divorced from a spouse, widowed, or never married. BMI measures (in kg/m^2^) were categorized using the following: <18.5 (thin), 18.5 to < 24 (normal), and ≥ 24 (overweight). Residence was defined either as urban or rural. Smoking and drinking habits were classified either as “never” or “current.”

#### Health Status

Health status included hypertension, dyslipidemia, diabetes, cancer, chronic lung diseases, heart problems, arthritis, asthma, falls, hip fractures, near- and far-vision impairment, hearing problems, eating frequency, depressive symptoms, and instrumental activities of daily living (IADLs). Eating frequency was classified either as eating three times per day, eating more than three times per day, or eating less than three times per day. Depressive symptoms were assessed using the 10-item Center for Epidemiologic Studies Short Depression (CES-D) Scale, wherein a score greater than or equal to 10 indicates a significant depressive symptom (Andresen et al., 1994). Performance in IADLs was examined using five items: household chores, cooking, shopping, managing money, and taking medications. Participants were categorized as impaired if they scored more than 5 on the questionnaire. Responses for other items were dichotomized either as “yes” or “no.”

#### Frailty and Other Physical Performance Test

Frailty was first described by Fried et al. (2001). It was measured using the physical frailty phenotype scale developed in the Cardiovascular Health Study and is objectively identified into five elements: weakness, SG, exhaustion, inactivity, and shrinking (Fried et al., 2001). Weakness was defined based on maximum grip strength of either hand, which is in the lowest 20th percentile of population distribution while adjusting for sex and BMI. SG was defined based on time to walk a 2.5-m course, which is in the lowest 20th percentile of population distribution while adjusting for sex and height. Exhaustion was identified if they answered “a moderate amount of time; 3–4 days” or “most of the time” to either of the two questions from the CES-D scale: “I could not get going” and “I felt everything I did was an effort.” Inactivity was defined within the lowest level of physical activity as measured by the International Physical Activity Questionnaire. Shrinking was defined either as self-reporting a loss of five or more kilograms in the previous year or a BMI of 18.5 kg/m^2^ or less. In summary, individuals with none were considered “non-frail”; meanwhile, those satisfying one or two criteria were considered “prefrail,” and those with at least three to five criteria were defined as “frail.” Lowest five chair stands were defined based on time to stand and sit five times as quickly as possible from the chair, which is in the lowest 20th percentile of population distribution while adjusting for sex and height. Lowest standing balance was defined if they were not able to maintain their feet in a side-by-side position for 10 s each.

#### MCR

MCR diagnosis was built with cognitive complaints but without significant functional impairment building on current operational definitions for mild cognitive impairment criteria (Petersen, 2011). As described above, it is a syndrome defined as SG combined with SCD. In our study, participants met the criteria for SCD if they answered “poor” to the following survey item: “How would you rate your memory at the present time? Would you say it is excellent, very good, good, fair, or poor?”

### Follow-Up and Outcome

Under CHARLS, participants were observed once every 2 years and during follow-up visits using a complete cognitive test. Cognitive performance was calculated using three categories: mental intactness, episodic memory, and global cognition (Liu et al., 2019). Mental intactness included numerical ability (serial sevens), time orientation (date, month, year, day of the week, and season), and picture drawing (intersecting pentagon copying test), with scores ranging from 0 to 11. Episodic memory included immediate and delayed word recall, with scores ranging from 0 to 20. Global cognition was scored using the summation of the episodic memory and mental intactness scores, which ranges from 0 to 31. The main study outcome was cognitive impairment, which was used to categorize whether participants were in the lowest 10% of the distribution of global cognition during follow-ups. Furthermore, this population-based 10% cutoff point is a sensitive and specific maker of cognitive impairment (Ganguli et al., 1993) and has been used in other studies (Chandra et al., 1998; Yaffe et al., 2001; Mulsant et al., 2003).

### Predictors Selection and Model Development

All 1,177 participants in the development cohort were included for predictor selection and model development. First, 35 variables were chosen into the selection process. least absolute shrinkage and selection operator (LASSO) method was applied to minimize the potential collinearity and overfitting of variables. Imputation for missing variables was considered if values were less than 20%. We used predictive mean matching and a dummy variable to impute numeric features and binary variables or factor features, respectively. The most useful predictors were selected using 1 standard error (1-SE) of the minimum criteria. The subsequent confidence interval (CI) identified by LASSO regression analysis was estimated using Kaplan–Meier method, and the log-rank test was used to compare survival curves. The Cox proportional hazard model was employed to develop a multivariate model and predict the 4-year cognitive impairment probability; meanwhile, a backward procedure based on Akaike information criterion (AIC) (Bozdogan, 1987) was used to construct the prediction model. The proportional hazards assumption was checked using graphical diagnostics based on scaled Schoenfeld residuals (Schoenfeld, 1982). To provide the clinician with a quantitative tool to predict individual probability of cognitive impairment, we built a multivariable Cox analysis–based nomogram in the development cohort.

### Performance and Internal Validation of the Nomogram in the Development Cohort

Calibration curves were plotted to assess the nomogram. The Harrell’s concordance index (C-index) was measured to evaluate the discrimination performance of the nomogram. To calculate a relatively corrected calibration and C-index, bootstrapping validation (1,000 bootstrap resamples) was performed to the nomogram.

### External Validation of the Nomogram

External validation was performed using the dataset from 2012 to 2016 in HRS. First, the total scores of each individual were calculated according to the constructed nomogram in the validation cohort. Second, we used the total scores as a factor to perform Cox regression analysis in this cohort, and finally, the C-index and calibration curve were derived based on the regression analysis.

### Clinical Use

Decision curve analysis was performed to determine the clinical usefulness of the nomogram by quantifying the net benefits at different threshold probabilities in the validation dataset (Vickers et al., 2008).

### Statistical Analysis

Results were presented as mean ± standard deviation or median (range) and number (percentage) for continuous variables and categorical variables, respectively. Continuous variables were explored for potential non-linear association with the risk of cognitive impairment using restricted cubic splines. LASSO Cox regression was done using the “glmnet” package. Multivariate Cox regression, nomograms, and calibration plots were done using the “rms” package. C-index calculation was performed using the “Hmisc” package. Internal validation was performed using the “rms” package. Decision curve analysis was performed with the “dca.R” function. The statistical significance levels for all analyses were two-sided, with a statistical significance of 0.05. Statistical analysis was performed with STATA version 15.0 (StataCorp, College Station, TX) and R software version 4.0.0^1^.

## Results

### Characteristics of Participants in the Development and Validation Cohorts

In the development cohort, 1,177 individuals (54.7% male) were included in the current study (Supplementary Figure 1). The median follow-up time was 48.0 months (range, 19.0–53.0 months) with a 4-year CI rate of 16.7%. The median age was 65.0 years (range, 60–90 years), and 17.6% of the participants reached higher education level. The median baseline cognition was 14.0, ranging from 6.0 to 28.0; 31.4% of the participants had SCD, through which 19.9% and 6.7% had SG and MCR, respectively. The characteristics of participants in the development cohorts are listed in Table 1. Similarly, the procedure and exclusion criteria are depicted in Supplementary Figure 1.

**TABLE 1A:** Characteristics of participants in the development and validation cohorts.

	Development cohort (*n* = 1,177)	Validation cohort (*n* = 2,076)
**Cognitive impairment**		
Yes	196 (16.7)	388 (18.7)
No	981 (83.3)	1,688 (81.3)
Follow-up time (months), median (range)	48.0 (19.0–53.0)	48.0 (14.0–63.0)
Age (years), median (range)	65.0 (60.0–90.0)	73.0 (65.0–97.0)
**Gender, no. (%)**		
Male	644 (54.7)	839 (40.4)
Female	533 (45.3)	1,237 (59.6)
**Education, no. (%)**		
High school or less (≤12 years)	970 (82.4)	1,009 (48.6)
College or higher (>12 years)	207 (17.6)	1,067 (51.4)
Baseline cognition, median (range)	14.0 (6.0–28.0)	24.0 (18.0–34.0)
**Subjective cognitive decline, no. (%)**		
Yes	369 (31.4)	55 (2.6)
No	808 (68.6)	2,021 (97.4)
**Slow gait, no. (%)**		
Yes	234 (19.9)	344 (16.6)
No	943 (80.1)	1,732 (83.4)
**Motoric cognitive risk syndrome**		
Healthy	653 (55.5)	1,693 (81.6)
Only subjective cognitive decline	290 (24.6)	39 (1.9)
Only slow gait	155 (13.2)	328 (15.8)
Motoric cognitive risk syndrome	79 (6.7)	16 (0.7)

### Predictors Selection and Development of an Individualized Prediction Model

**FIGURE 1:**
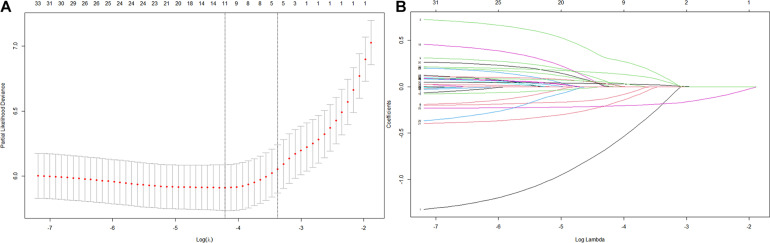
Variables selection using the least absolute shrinkage and selection operator (LASSO) Cox regression model. **(A)** Tuning parameter (λ) selection in the LASSO model used 10-fold cross-validation via 1 standard error (SE) of the minimum criteria (the 1-SE criteria). **(B)** LASSO coefficient profiles of the 35 variables.

**FIGURE 2:**
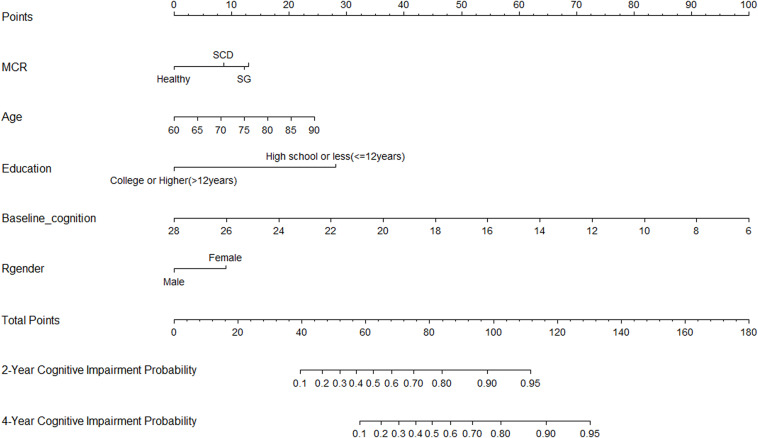
Nomogram for predicting the 4-year cognitive impairment probability. To calculate the cognitive impairment probability for a specific patient, locate patient’s MCR status, and draw a line straight upward to the Points axis to determine the score associated with that status. Repeat the process for age, education status, baseline cognition, and gender; sum the scores for each factor; and locate this sum on the Total Points axis. Then, draw a line straight down to the corresponding 2- or 4-year cognitive impairment probability axis to find the predicted cognitive impairment probability.

Thirty-five variables were included in the LASSO regression. After LASSO regression selection (Figures 1A,B), five variables remained significant predictors of cognitive impairment, which included education, gender, baseline cognition, age, and MCR. Furthermore, we found that there was sufficient evidence for a linear relationship between age, baseline cognition, and risk of cognitive impairment. Furthermore, inclusion of these five variables in a Cox regression model resulted to independently statistically significant predictors of cognitive impairment for all variables. Hence, these were included in final prediction model after a backward procedure based on AIC. These variables included the following: MCR (HR, 1.952; 95% CI, 1.205–3.160; *P* = 0.007), education (HR, 0.907; 95% CI, 0.879–0.936; *P* < 0.001), gender (female vs. male) (HR, 1.568; 95% CI, 1.166–2.110; *P* = 0.003), age (HR, 1.042; 95% CI, 1.019–1.065; *P* < 0.001), and baseline cognition (HR, 0.792; 95% CI, 0.758–0.828; *P* < 0.001) (Table 2). The nomogram that integrated all significant independent factors for cognitive impairment in the development cohort is shown in Figure 2. Similarly, the results of the univariate analysis are listed in Supplementary Table 1.

**TABLE 1B:** Multivariable Cox regression model for predicting development of cognitive impairment in 1,177 participants.

Independent variable	Cognitive impairment HR (95% CI)	*P-*value
**MCR**		
**Healthy**	**Ref.**	
Subjective cognitive decline	1.564 (1.121–2.183)	0.009
Slow gait	1.842 (1.205–2.817)	0.005
Motoric cognitive risk syndrome	1.952 (1.205–3.160)	0.007
Age	1.042 (1.019–1.065)	<0.001
Education	0.907 (0.879–0.936)	<0.001
**High school or less (≤12 years)**	**Ref.**	
College or higher (>12 years)	0.232 (0.094–0.570)	0.001
Baseline cognition	0.792 (0.758–0.828)	<0.001
Gender (female vs. male)	1.568 (1.166–2.110)	0.003

**CI, confidence interval; HR: hazard ratio; Ref., reference.**

### Performance and Internal validation of Nomogram in the Development Cohort

**FIGURE 3:**
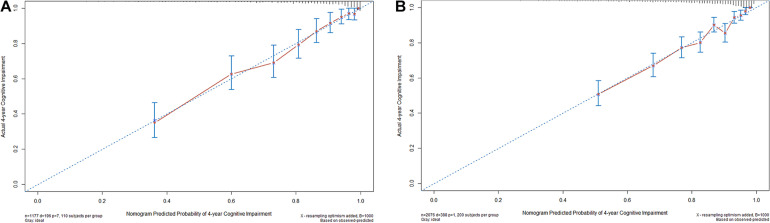
The internal **(A)** and external **(B)** calibration curves of the nomogram. Nomogram-predicted probability and observed frequency over 4 years for cognitive impairment among participants with normal cognition at baseline were plotted in the *x*- and *y*-axis, respectively. The gray line indicates the ideal plot for the calibration curve, where the nomogram-predicted probabilities perfectly match the observed probabilities in all subgroups.

The nomogram was internally validated using the bootstrap-corrected calibration plot and Harrell C statistic, which resulted to a Harrell C-index for cognitive impairment prediction of 0.814 (95% CI, 0.782–0.835). The bootstrap-corrected calibration plot for the probability of 4-year survival showed an optimal agreement between the prediction by nomogram and actual observation (Figure 3A).

### Comparison of Predictive Accuracy Between the Nomogram and MCR

A reference model based on MCR alone yielded a C-statistic of 0.615 (95% CI, 0.571–0.645), which is significantly worse as compared with prediction nomogram (*P* < 0.001).

### External Validation of Predictive Accuracy of the Nomogram for Cognitive Impairment

For the validation cohort, we studied 2,076 individuals (Supplementary Figure 2). The median follow-up time was 48.0 months (range, 14.0–63.0 months), with 18.7% of participants suffering from cognitive impairment during follow-up. The baseline characteristics of the CHARLS and HRS participants were comparable, whereas the HRS participants were older and more educated and reported less SCD, slower gait, and MCR on average (Table 1). The Harrell C-index of the nomogram for predicting cognitive impairment was 0.772 (95% CI, 0.776–0.818), and the calibration curve showed good agreement between prediction and observation in the 4-year cognitive impairment probability (Figure 3B).

### Clinical Use

The decision curve analysis for the nomogram is presented in Supplementary Figure 3. The net benefit curves for cognitive impairment over 4 years show that there is higher net benefit than strategies based on considering either no participants or all participants for intervention at risk thresholds up to approximately 80%.

## Discussion

We developed and validated a prediction nomogram for cognitive impairment in cognitively healthy older adults. The nomogram incorporates five items: MCR, age, education, baseline cognition, and gender. The nomogram can be used to calculate the 4-year risk of cognitive impairment and successfully stratified participants according to their individual risks. All predictors included in the nomogram were easy to assess and readily available. There were also no additional expensive tests needed, such as brain imaging. Incorporating the MCR and other risk factors into an easy-to-use nomogram facilitated the preoperative individualized prediction of cognitive impairment.

The combined analysis of a prediction nomogram model based on MCR and other important risk factors, rather than individual components, resulted in better predictive performance to identify cognitive impairment. MCR syndrome provides incremental validity over its individual components (Verghese et al., 2014). However, age, educational attainment, baseline cognition, and gender are also strongly associated with risk of cognitive impairment. Although MCR has previously been described by researchers for a screening marker of cognitive impairment, our study has some important differences from this previous work. In this analysis, we estimated 4-year risk of cognitive impairment and incorporated these into a simple point-scoring scheme for predicting cognitive impairment risk that has significant practical utility.

Previous studies have investigated MCR (Sonnega et al., 2014; Verghese et al., 2019) or frailty (Panza et al., 2019; Wallace et al., 2019) as biomarkers of cognitive impairment and dementia in cognitively healthy older adults. However, this study noted that frailty showed enough predictive strength on the basis of univariable association with cognitive impairment but not included in the prediction nomogram; however, the rejection of important predictors may be a result of collinearity or confounding by other predictors (Collins et al., 2015). As a qualitative prediction model, MCR can be easily measured. Our study further demonstrated that MCR was associated with cognitive impairment, and the MCR-based nomogram was identified as a useful tool in the selection of high-risk patients for early intervention studies and applications of personalized medicine.

For the prediction nomogram, 35 variables were reduced to five predictors by shrinking the regression coefficients with the LASSO method. This method not only surpasses the method of choosing predictors on the basis of the strength of their univariable association with outcome (Boyd, 2005), but also enables the panel of selected features to be combined into a prediction nomogram. Multimarker analyses that incorporate individual markers into marker panels have been incorporated in recent studies (Exalto et al., 2013; Downer et al., 2016; Li et al., 2018; Licher et al., 2019; Zhou et al., 2020). Similarly, the prediction nomogram that combined multiple individual risk factors demonstrated adequate discrimination in the development cohort (C-index, 0.814; 95% CI, 0.782–0.835); meanwhile, maintenance was observed in the validation cohort (C-index, 0.772; 95% CI, 0.776–0.818). The opportunity to undertake an external validation in HRS, which was conducted in a different geographical location, corroborated our findings. More importantly, different predictors were distributed in the development and validation cohort, which it only slightly affected the performance of nomogram in the validation cohort, emphasizing that the nomogram is robust.

Given that incidence of cognitive impairment was comparable in the two cohorts, the nearly equal discrimination demonstrated that the prediction nomogram was stable for prediction and could be applied directly in the validation cohort. This involved omitting the process of adjusting intercept and regression coefficients regarding the nomogram construction, as well. In a recent study that investigated the dementia risk of using age, history of stroke, SCD, and need for assistance with finances or medication, the derived accuracy of combined risk factor was 78%. This is lower than the C-index of the prediction nomogram we constructed. The most important and final dispute for the use of the nomogram was based on the need to interpret individual need of additional intervention. However, the performance of nomogram based on MCR, discrimination, and calibration could not capture the clinical consequences of a particular level of discrimination or degree of miscalibration (Localio and Goodman, 2012; Collins et al., 2015; Van Calster and Vickers, 2015). Therefore, we assessed whether decisions based on the nomogram would improve individual outcomes to justify the clinical usefulness. As a result, decision curve analysis was applied in this study, and it offers insights into outcomes based on threshold probability, from which the net benefit could be derived. The decision curve showed that if the threshold probability of a patient or doctor is 0–80%, using the prediction nomogram in the current study to predict cognitive impairment adds more benefit than either the treat-all-patients schedule or the treat-none schedule.

Study limitations include the non-consideration of environmental risk factors or genetic risk factors on the assessment of 35 candidate predictors. However, it is yet to be decided whether simply building a model that applies the easy-to-get features to predict outcomes directly is preferable to combined genetic factors. Second, we used a regularization method (LASSO) that automatically selects and subsequently shrinks effect sizes of important predictors. This may have led to some underestimation of predictor effects in the development data.

## Conclusion

In conclusion, this study presented a nomogram that incorporated both MCR and other risk factors and can be conveniently used to facilitate the preoperative individualized prediction of cognitive impairment in cognitively healthy older adults.

## Data Availability Statement

The original contributions presented in the study are included in the article/Supplementary Material, further inquiries can be directed to the corresponding author/s.

## Ethics Statement

Ethics approval for data collection in Charls was obtained from the Biomedical Ethics Review Committee of Peking University (IRB00001052-11015). The participants provided their written informed consent to participate in this study.

## Author Contributions

YL and CL designed this study and drafted the manuscript. YL and KW acquired the data. YL, LJ, and NG performed the statistical analysis, assisted by LF and CL. KW and LF reviewed the manuscript. All authors approved the final version for submission.

## Conflict of Interest

The authors declare that the research was conducted in the absence of any commercial or financial relationships that could be construed as a potential conflict of interest.

## Footnotes

1 http://www.r-project.org
