# Supplementary material for: Development and Validation of a Nomogram Based on Motoric Cognitive Risk Syndrome for Cognitive Impairment
Source: Front Aging Neurosci. 2021 Apr 16;13:618833. doi: 10.3389/fnagi.2021.618833 (PMC8086554; doi:10.3389/fnagi.2021.618833)
Supplement: Supplementary file 1 [file Table_1.DOCX]

Supplementary Material

**Table 1. Univariate analysis of baseline characteristics on cognitive impairment**

|  | **variables** | **HR (SE)** | **95% CI** |
| --- | --- | --- | --- |
| **Cognitive impairment** | Baseline global cognition | **0.764(0.017)^***^** | **0.732- 0.798** |
|  | Age | **1.067(0.013)^***^** | **1.042- 1.091** |
|  | SCD | **2.061(0.295)^***^** | **1.557-2.728** |
|  | Lowest SG | **1.616(0.257)^**^** | **1.183-2.207** |
|  | MCR(SCD vs healthy) | **2.462(0.415)^***^** | **1.769- 3.425** |
|  | MCR(SG vs healthy) | **2.160(0.457)^***^** | **1.427-3.272** |
|  | MCR(MCR vs healthy) | **2.607(0.638)^***^** | **1.613-4.213** |
|  | Lowest standing balance | **1.698(0.259)^**^** | **1.260-2.289** |
|  | Weight loss | 1.088(0.233) | 0.714-1.655 |
|  | Lowest GS | **1.802(0.279)^***^** | **1.330-2.442** |
|  | Lowest CS | **1.581(0.250)^**^** | **1.160-2.156** |
|  | Lowest physical activity | 1.272(0.223) | 0.902-1.795 |
|  | Exhaustion | **1.617(0.309)^*^** | **1.112- 2.350** |
|  | Frailty(pre-frailty vs healthy) | **1.608(0.257)^**^** | **1.175-2.200** |
|  | Frailty(frailty vs healthy) | **2.646(0.573)^***^** | **1.730- 4.045** |
|  | IADL(unimpaired) | **0.684(0.120)^*^** | **0.484- 0.965** |
|  | CES(non-depressed) | 0.891(0.133) | 0.665-1.194 |
|  | BMI(thin vs normal) | 1.567(0.375) | 0.981-2.504 |
|  | BMI(overweight vs normal) | 0.874(0.135) | 0.645-1.183 |
|  | Residence (urban versus rural) | **0.512(0.086)^***^** | **0.369-0.710** |
|  | Educational attainment(high school or less vs college or higher) | **0.114(0.052)^***^** | **0.047-0.277** |
|  | Marital status (married) | **0.570(0.095)^**^** | **0.412-0.790** |
|  | Gender (female vs male) | **2.041(0.300)^***^** | **1.531-2.723** |
|  | Hypertension | 1.112(0.170) | 0.823-1.501 |
|  | Dyslipidemia | 0.912(0.221) | 0.568-1.467 |
|  | Diabetes | 0.648(0.234) | 0.319-1.135 |
|  | Cancer | 2.385(1.206) | 0.886-6.426 |
|  | Lung Diseases | 0.768(0.190) | 0.473-1.248 |
|  | Heart problems | 1.022(0.215) | 0.676-1.544 |
|  | Arthritis | 1.204(0.177) | 0.903-1.606 |
|  | Asthma | 0.809(0.312) | 0.381-1.721 |
|  | Falls | **1.431(0.247)^*^** | **1.020-2.007** |
|  | Hip fractures | 2.231(1.127) | 0.829-6.005 |
|  | Near-vision impairment | 0.988(0.179) | 0.693-1.408 |
|  | Far-vision impairment | **1.382(0.220)^*^** | **1.012-1.887** |
|  | Hearing problems | 1.029(0.249) | 0.641-1.652 |
|  | Eating frequency (3 vs more than 3) | 0.640(0.247) | 0.300-1.363 |
|  | Eating frequency (less than 3 vs more than 3) | 0.841(0.358) | 0.365-1.938 |
|  | Smoking | 0.837(0.122) | 0.629-1.114 |
|  | Drinking | 0.903(0.150) | 0.652-1.250 |

*Note.* β: beta coefficient, SE: standard error. SCD: subjective cognitive decline; SG: slow gait; MCR: motoric cognitive risk syndrome; GS: grip strength; CS: chair stand; IADL: instrumental activities of daily living; CES-D: Center for Epidemiologic Studies Short Depression Scale; BMI: Body mass index

^*^p <0.05, ^**^ p <0.005, ^***^ p <0.001.


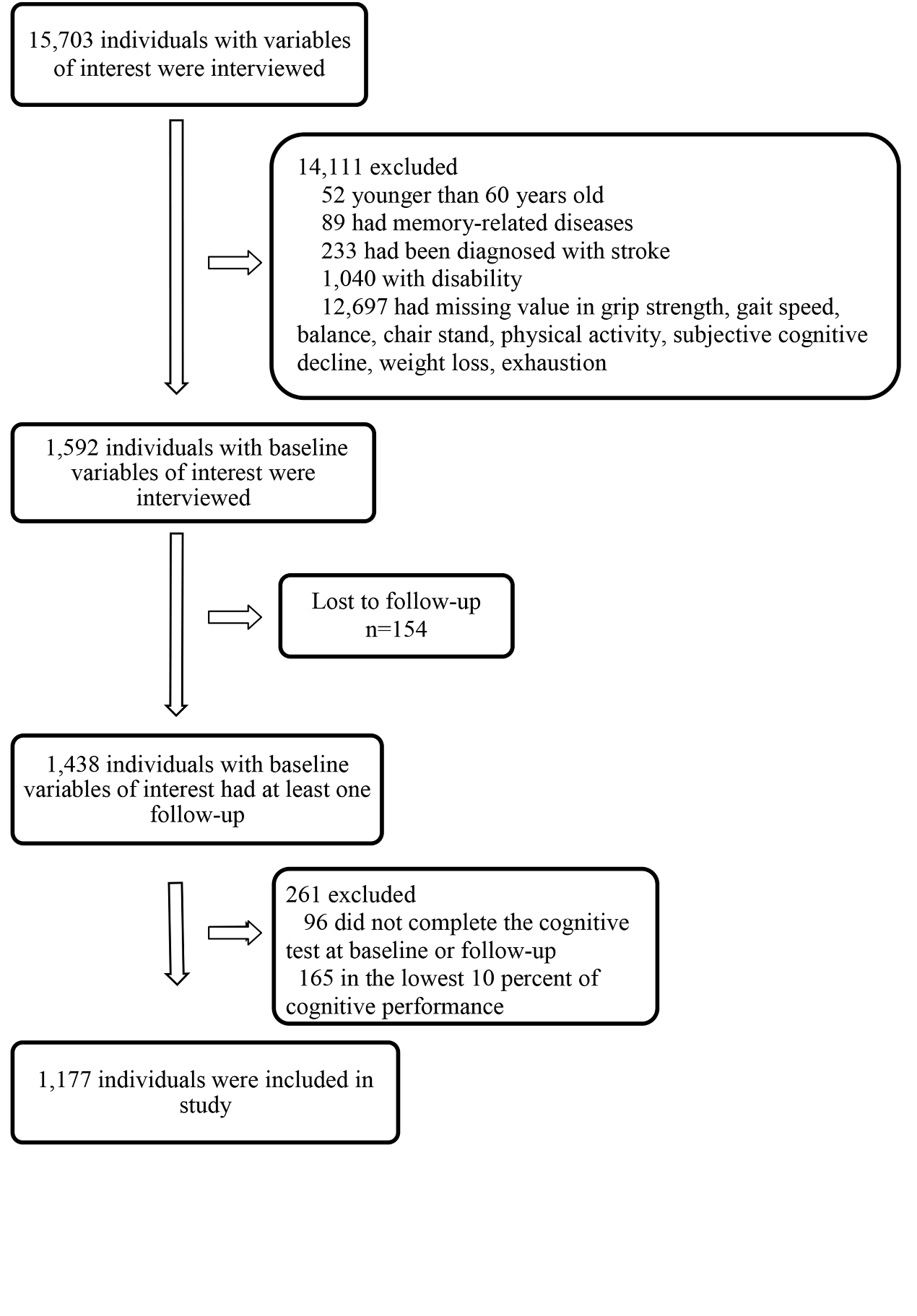
Figure 1. Cohort selection criteria for the development cohort, CHARLS Survey from 2011 to 2015


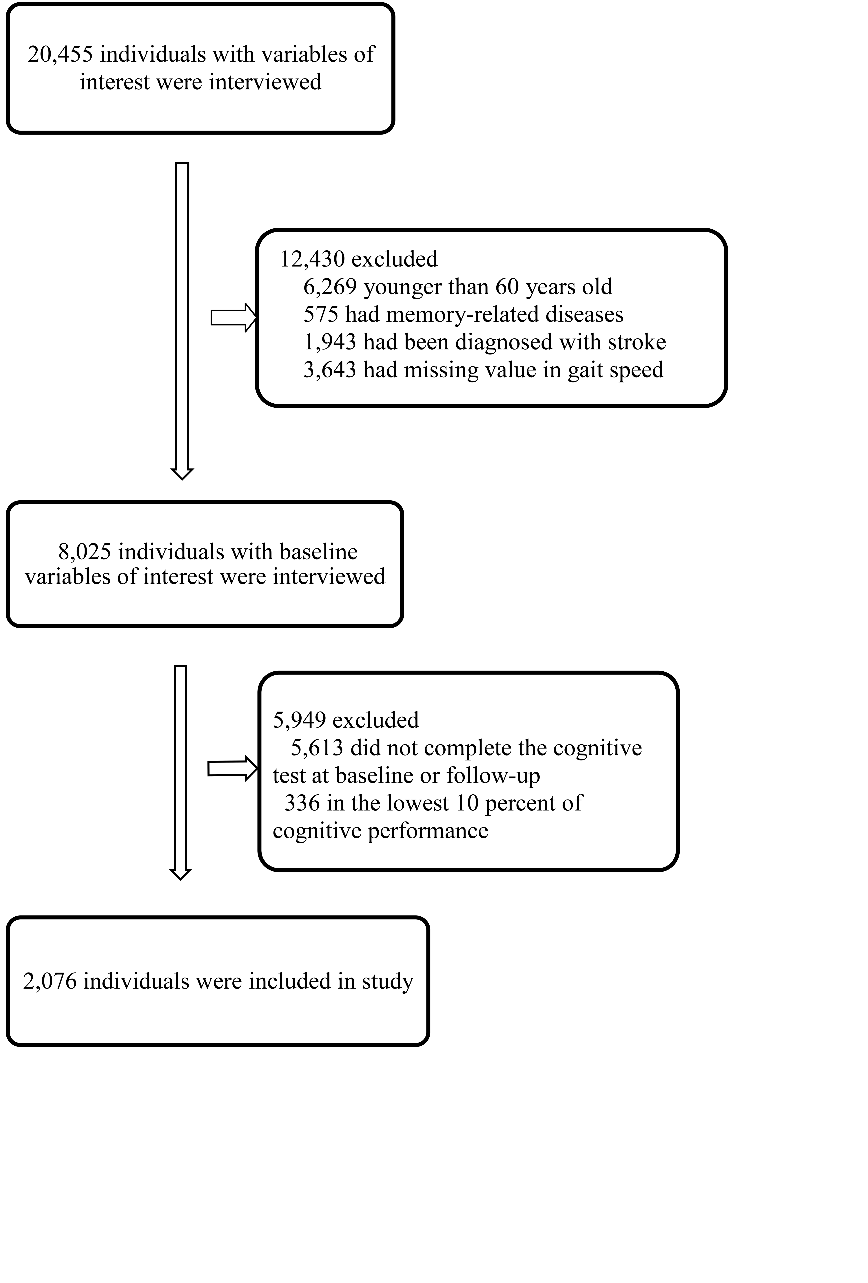


Figure 2. Cohort selection criteria for the validation cohort, HRS Survey from 2012 to 2016


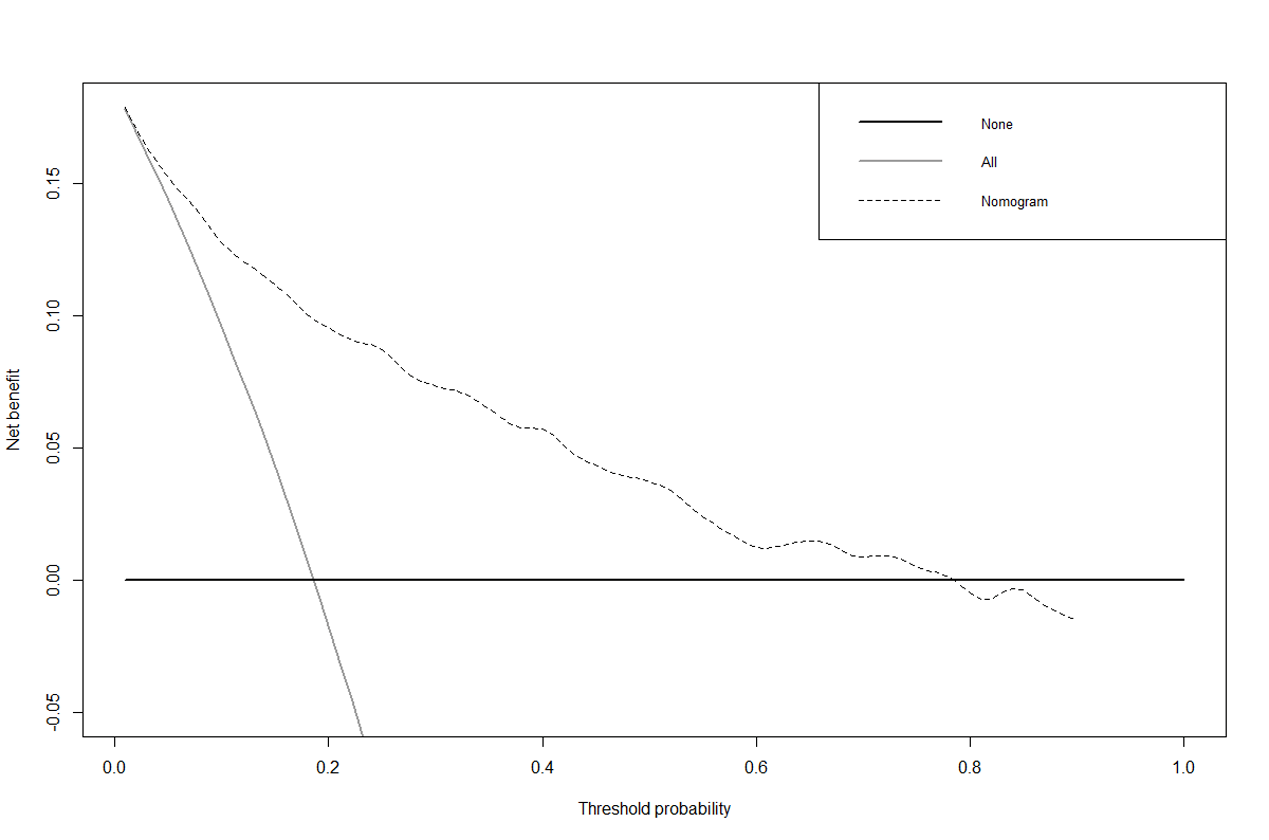


Figure 3. Decision curve analysis for nomogram
